# Neutrophil Extracellular Traps Induced by Shiga Toxin and Lipopolysaccharide-Treated Platelets Exacerbate Endothelial Cell Damage

**DOI:** 10.3389/fcimb.2022.897019

**Published:** 2022-06-23

**Authors:** Verónica Inés Landoni, Jose R. Pittaluga, Agostina Carestia, Luis Alejandro Castillo, Marcelo de Campos Nebel, Daiana Martire-Greco, Federico Birnberg-Weiss, Mirta Schattner, Pablo Schierloh, Gabriela C. Fernández

**Affiliations:** ^1^ Laboratorio de Fisiología de los Procesos Inflamatorios, Instituto de Medicina Experimental (IMEX)-Consejo Nacional de Investigaciones Científicas y Técnicas (CONICET)/Academia Nacional de Medicina de Buenos Aires, Ciudad Autónoma de Buenos Aires (CABA), Argentina; ^2^ Laboratorio de Trombosis Experimental e Inmunobiología de la Inflamación, Instituto de Medicina Experimental (IMEX)-Consejo Nacional de Investigaciones Científicas y Técnicas (CONICET)/Academia Nacional de Medicina de Buenos Aires, Ciudad Autónoma de Buenos Aires (CABA), Argentina; ^3^ Laboratorio de Mutagénesis, Instituto de Medicina Experimental (IMEX)-Consejo Nacional de Investigaciones Científicas y Técnicas (CONICET)/Academia Nacional de Medicina de Buenos Aires, Ciudad Autónoma de Buenos Aires (CABA), Argentina; ^4^ Instituto de Investigación y Desarrollo en Bioingeniería y Bioinformática, Centro Científico Tecnológico Consejo Nacional de Investigaciones Científicas y Técnicas (CONICET), Santa Fe, Argentina

**Keywords:** endothelial cell damage, netosis, platelets, Shiga toxin, LPS

## Abstract

Hemolytic uremic syndrome (HUS) is the most common cause of acute renal failure in the pediatric population. The etiology of HUS is linked to Gram-negative, Shiga toxin (Stx)-producing enterohemorrhagic bacterial infections. While the effect of Stx is focused on endothelial damage of renal glomerulus, cytokines induced by Stx or bacterial lipopolysaccharide (LPS) and polymorphonuclear cells (PMNs) are involved in the development of the disease. PMN release neutrophil extracellular traps (NETs) to eliminate pathogens, although NETs favor platelets (Plts) adhesion/thrombus formation and can cause tissue damage within blood vessels. Since thrombus formation and occlusion of vessels are characteristic of HUS, PMN–Plts interaction in the context of Stx may promote netosis and contribute to the endothelial damage observed in HUS. The aim of this study was to determine the relevance of netosis induced by Stx in the context of LPS-sensitized Plts on endothelial damage. We observed that Stx2 induced a marked enhancement of netosis promoted by Plts after LPS stimulation. Several factors seemed to promote this phenomenon. Stx2 itself increased the expression of its receptor on Plts, increasing toxin binding. Stx2 also increased LPS binding to Plts. Moreover, Stx2 amplified LPS induced P-selectin expression on Plts and mixed PMN–Plts aggregates formation, which led to activation of PMN enhancing dramatically NETs formation. Finally, experiments revealed that endothelial cell damage mediated by PMN in the context of Plts treated with LPS and Stx2 was decreased when NETs were disrupted or when mixed aggregate formation was impeded using an anti-P-selectin antibody. Using a murine model of HUS, systemic endothelial damage/dysfunction was decreased when NETs were disrupted, or when Plts were depleted, indicating that the promotion of netosis by Plts in the context of LPS and Stx2 plays a fundamental role in endothelial toxicity. These results provide insights for the first time into the pivotal role of Plts as enhancers of endothelial damage through NETs promotion in the context of Stx and LPS. Consequently, therapies designed to reduce either the formation of PMN–Plts aggregates or NETs formation could lessen the consequences of endothelial damage in HUS.

## Introduction

The epidemic form of hemolytic uremic syndrome (HUS) occurs after a prodrome of hemorrhagic colitis caused by Shiga toxin (Stx)-producing *Eschericchia coli* organisms (STEC) (Mele et al., 2014). HUS is the leading cause of acute renal failure in children, which goes together with hemolytic anemia and thrombocytopenia (Karpman et al., 2010; Talarico et al., 2016). Endothelial dysfunction at the level of the entire vasculature is extremely important in this disease, and it is a determinant factor in the sequence of events that leads to the microangiopathic process observed in HUS. Endothelial damage occurs through the interaction of Stx with its globotriaosylceramide (Gb_3_) receptor (Proulx et al., 2001). Although Stx is the main pathogenic factor for HUS development, the inflammatory response can potentiate Stx toxicity. Both bacterial lipopolysaccharide (LPS) and polymorphonuclear neutrophils (PMN) play an important role in the full development of HUS (Exeni et al., 2007; Lee and Tesh, 2019). In fact, neutrophilia correlates with poor prognosis in HUS patients (Milford et al., 1991; Fernandez et al., 2007).

Neutrophil extracellular traps (NETs) are networks of extracellular decondensed chromatin, which contain histones, granule-derived enzymes, and several cytoplasmic proteins (Brinkmann et al., 2004). NETs are produced to allow neutrophils to trap and kill microbes in the extracellular environment (Brinkmann et al., 2004). However, NETs may play pathogenic roles in several conditions including atherosclerosis (Knight et al., 2014), rheumatoid arthritis (Apel et al., 2018), systemic vasculitis (Kessenbrock et al., 2009), and systemic lupus erythematosus (Villanueva et al., 2011). In particular, NETs have been also associated with HUS complications (Ramos et al., 2016; Leffler et al., 2017).

Although initially it was proposed that NETs are formed within tissues at the site of infection, the formation of NETs (netosis) inside blood vessels has also been observed. In this sense, the detrimental effect of NETs in the peripheral vasculature when Stx reaches the circulation and tissues is a matter of speculation. On the other hand, the interrelationship between the inflammatory and thrombotic responses has been demonstrated by numerous works. In fact, it has been determined that the interaction between PMN and platelets (Plts) induces activation of PMN, and Plts treated with high doses of LPS are capable of inducing netosis (Clark et al., 2007; Liu et al., 2016). At the same time, NETs bind Plts and support their aggregation, indicating that they are substrates for Plts adhesion and also provide a stimulus for Plts activation promoting thrombosis (Fuchs et al., 2010).

The literature reports activation of endothelial cells, PMN, and Plts by Stx, although these effects are exacerbated in the presence of other inflammatory components, such as LPS (Van de Kar et al., 1992; Van Setten et al., 1997; Karpman et al., 2001; Ståhl et al., 2009; Arfilli et al., 2010; Brigotti et al., 2010; Brigotti et al., 2013). Moreover, the effects of Stx on Plts, endothelium or PMN have been reported individually, or in combinations between two of these components, but studies integrating the three cell types are missing. This is crucial to fully comprehend the thrombotic/endothelial damage observed in HUS patients and to integrate NETs into the pathophysiological scenario of HUS.

Given that occlusion by thrombi within blood vessels and endothelial damage/dysfunction is typical in HUS, the hypothesis of this work is that Stx2 and LPS trigger the interaction of PMN with Plts promoting NETs formation inside blood vessels, playing a role in the endothelial damage observed in HUS. Therefore, this work aimed to determine the relevance of netosis promoted by Ptls in the context of Stx2 and LPS on endothelial cell damage.

## Materials and Methods

### Ethics Statement

Human platelets (Plts) and neutrophils (PMN) were isolated from healthy adult blood donors in agreement with the guidelines of the Ethical Committee of Academia Nacional de Medicina de Buenos Aires. All adult blood donors provided their informed consent prior to the study in accordance with the Declaration of Helsinki (2013) of the World Medical Association.

### Human Donors

Human normal blood samples were obtained from voluntary donors by venipuncture and drawn directly into plastic tubes containing 3.8% sodium citrate.

### Endothelial Cells

The human microvascular endothelial cell 1 line (HMEC-1) was used. Cells were maintained in MCDB 131 (Gibco, Thermo Fisher Scientific, Waltham, MA, USA) supplemented with 10% fetal bovine serum (FBS) (Gibco, Thermo Fisher Scientific, Waltham, MA, USA), epithelial growth factor (10 ng ml^−1^), hydrocortisone (1 µg ml^−1^), L-glutamine (10 mM), penicillin (100 U ml^−1^), and streptomycin (100 μg ml^−1^) (Sigma-Aldrich, MI, USA), and incubated at 37°C in 5% CO2.

### PMN Purification and Plts Preparation

Human PMN were isolated by Ficoll–Hypaque gradient centrifugation (Ficoll Pharmacia, Uppsala; Hypaque, Wintthrop Products, Buenos Aires, Argentina) and dextran sedimentation, as previously described (Böyum, 1968). Viability was assessed by trypan blue exclusion, and purity was determined by Turk’s solution staining. Only cell suspensions containing 98% of PMN were used.

Platelet-rich plasma (PRP) was obtained by centrifugation of blood samples (180*g* for 10 min). For washed Plts suspensions, PRP was centrifuged in the presence of prostacyclin (PGI_2_, 75 nM), and Plts were then washed in washing buffer (140 mM NaCl, 10 mM NaHCO_3_, 2.5 mM KCl, 0.5 mM Na_2_HPO4, 1 mM MgCl_2_, 22 mM sodium citrate, 0.55 mM glucose, 0.35% BSA, pH 6.5). Washed Plts were resuspended in Tyrode’s buffer, and the Plts number was adjusted. In experiments where co-culture of PMN and Plts were used, cells were obtained from the same donor.

### Experimental Design

Purified recombinant Shiga toxin type 2 (Stx2) was purchased from Tufts University (Boston, MA, USA). It contained <5 pg of LPS (per μg of Stx), quantified by the *Limulus amebocyte* lysate assay. Commercially purified LPS from *E. coli* O111:B4 (Sigma-Aldrich, MI, USA) was used.

PMN or Plts were cultured in a medium with 3% FBS alone (Control) or in the presence of 20 ng ml^−1^ of Stx2 for 20 min before adding or not adding 0.3 µg ml^−1^ of LPS for another 20 min.

For PMN–Plts co-culture experiments, untreated or treated Plts were washed twice with a culture medium with 3% FBS to remove the stimuli before incubation with PMN for the indicated time depending on the assay. A ratio of PMN/Plts of 1:50 was used. In some experiments, an anti-P-selectin blocking antibody (1/50 dilution) (BD Biosciences, San José, CA, USA) was added to isolated Plts 5 min before co-culturing them with PMN.

In order to establish the specific effects of Stx2, an anti-Stx2 antibody (Toxin Technology, Sarasota, FL, USA) was used in some experiments. Stx2 was pre-incubated for 30 min with the anti-Stx2 antibody (1 µg/ml) and then was added where indicated.

### Neutrophil Extracellular Traps Formation

Neutrophil extracellular traps (NETs) were determined on 0.25×10^6^ PMN. Cells were treated or untreated with Stx2 and/or LPS, or in the presence of non-stimulated (washed) or treated Plts as described above (PMN/Plts ratio, 1:50). Treated PMNs were left for 6 h, and NETs were measured using different approaches as detailed below. Micrococcal Nuclease S7 (4 U, Hoffmann-La Roche, Basilea, Suiza) was used as a control to digest NETs.

#### Confocal Microscopy Determination of NETs

Cells were seeded gently onto glass coverslips coated with 0.001% poly-L-lysine in a 24-well plate in triplicate, allowed to settle, and incubated for 6 h at 37°C, 5% CO_2_. After the incubation period, samples were gently fixed with 4% paraformaldehyde, permeabilized with 0.25% Triton X-100, washed, and blocked with 3% BSA for 1 h. DNA was stained with propidium iodide (Vector Laboratories, Burlingame, CA, USA) and elastase using a specific anti-human neutrophil elastase antibody (Merk Millipore, Darmstadt, Germany). Plts were stained using anti-CD61 (BD Biosciences, San José, CA, USA), or the corresponding Ig isotype controls were used for setting non-specific binding (BD Biosciences, San José, CA, USA). Images for NET evaluation were acquired using a FluoView FV1000 confocal microscope (Olympus, Tokyo, Japan) equipped with a Plapon 60×/1.42 objective lens and processed using Olympus. At least 10 different fields were observed in each triplicate (200×). NET areas were determined as previously reported in at least five pictures obtained in 200× (Rodriguez-Rodrigues et al., 2017), using the wand tool from the FIJI software (Schindelin et al., 2012). The scale for the measurement was obtained from the data given in the confocal microscope image.

#### DNA–Protein Complexes Determination of NETs by ELISA

DNA–histone complexes were measured using a commercial kit according to manufacturers’ instructions (Cell Death Detection ELISA, Hoffmann-La Roche, Basilea, Suiza).

Elastase–DNA complexes were performed as previously described (Caudrillier et al., 2012). Briefly, 96-well plates were coated with 5 mg ml^−1^ anti-elastase antibody (Calbiochem), overnight at 4°C. After washing, plasma was added to the wells with incubation buffer containing a peroxidase-labeled anti-DNA antibody (dilution, 1:25) (Cell Death ELISA PLUS, Hoffmann-La Roche, Basilea, Suiza). The plate was then incubated for 2 h in continuous shaking at room temperature. After washing, peroxidase substrate (ABTS) (Thermo Fisher Scientific, Waltham, MA, USA) was added. Absorbance at 405 nm was measured after 20 min incubation at room temperature in the dark. Values for soluble elastase–DNA complexes were expressed as their fold increase in absorbance above control (untreated mice).

### Stx2 Receptor Expression

Globotriaosylceramide (Gb_3_ or CD77) expression was evaluated as previously described (Landoni et al., 2012). Briefly, untreated or treated Plts were fixed with 1% PFA and blocked with 3% BSA for 20 min and then exposed to a rat anti-CD77 monoclonal antibody (clone 38-13; final dilution, 1∶100; Immunotech Laboratories, Inc., Monrovia, CA, USA) for 45 min at 4°C. Then, Plts were washed and incubated with a fluorescein isothiocyanate (FITC)-conjugated secondary goat antibody F(ab)′_2_ anti-rat μ chain (final dilution 1∶200, Jackson Immuno Research Lab) in the dark for 30 min at 4°C. Immunostained cells were then washed and immediately analyzed by flow cytometry. Isotype matched control immunostaining was performed in parallel.

### Stx2 Association Analysis

Treated Plts were fixed and permeabilized with 2% paraformaldehyde containing 0.25% Triton X-100 for 20 min at room temperature in order to determine total Stx2 association (both bound and internalized). After blocking (3% BSA and 0.25% Triton X-100 in PBS) for 1 h at 4°C, samples were incubated for 2 h with mouse IgG1 anti-Stx (1:200 dilution; Toxin Technology, Sarasota, FL) or the corresponding isotype control antibody. Plts were then incubated with a secondary FITC anti-mouse antibody (1:100 dilution; Bio-Rad Laboratories, Hercules, CA) for 40 min at 4°C. Samples were analyzed by flow cytometry, and the association of Stx2 was determined by normalizing the mean fluorescence intensity (MFI) of treated Plts to the MFI binding of untreated Plts.

### LPS Binding Determination

The binding of LPS to Plts was determined using an FITC-labeled LPS (fluorescein isothiocynanate LPS 0111:B4 serotype, Sigma-Aldrich, MI, USA). FITC-labeled LPS (2.5 µg ml^−1^) was added to untreated or Stx2 (20 ng ml^−1^)-treated Plts for 40 min at 37°C. In a duplicated sample, a high dose of unlabeled LPS (10 µg ml^−1^) was added to treated Plts to compete and displace FITC-LPS bound to Plts and establish unspecific/soluble FITC that may have associated unspecifically to Plts. LPS association was determined by flow cytometry by normalizing the MFI of LPS-FITC-treated Plts to the MFI of unlabeled LPS-treated Plts.

### Evaluation of Cell Activation

To evaluate Plts and PMN activation, P-selectin and CD11b expression, respectively, were determined by direct immunofluorescence using conjugated anti-human monoclonal antibodies, and the surface expression of these activation markers was evaluated by flow cytometry. Plts were treated as indicated above, fixed, and stained with a FITC-conjugated anti-CD62P (anti-P-selectin, BD Biosciences, San José, CA, USA). For CD11b expression, PMN were stimulated with washed treated Plts for 40 min at 37°C and stained with a PE-conjugated anti-CD11b (Immunotech Laboratories, Inc., Monrovia, CA, USA).

### Platelet–Neutrophil Mixed Aggregate Assays

Isolated human treated Plts were incubated together with purified PMN in Tyrode’s buffer for 10 min, followed by staining with anti-human PE-CD11b and FITC-CD61 antibodies. For *in vivo* experiments, blood was obtained from mice, cells were stained using an anti-mouse FITC-CD61 (BD Biosciences, San José, CA, USA) and PE-Ly-6G (BioLegend, San Diego, CA, USA), and erythrocytes were lysed by a hypotonic shock of 15 s. Cells were washed and fixed. The results were expressed as the percentage of double-positive events within the PMN (CD11b for human or Ly-6G for mice)-gated region. In all cases, controls of isotype-matched antibodies were assayed in parallel.

### PMN-Mediated Cytotoxicity

Plts were treated and mixed with PMN without washing. Aggregates were seeded onto HMEC-1 monolayers (PMN/HMEC-1 ratio, 20∶1). After 18 h, non-adherent Plts/PMN and detached dead HMEC-1 were removed by vigorous washing. Remnant viable cells on wells were stained with violet crystal. To assess PMN-mediated HMEC-1 cytotoxicity, three random fields per treatment were photographed under 600× magnifications (Nikon, Coolpix 4500 digital camera; Nikon, Eclipse TE2000-S, inverted microscope). Cell counts were performed as previously described (Landoni et al., 2010) using ImageJ software (US National Institutes of Health, Bethesda, MD). The percentage of cytotoxicity was calculated as follows:


% Cytotoxicity=100−[(number of treated HMEC−1number of untreated HMEC−1)×100]

*IL-8 and IL-6 Determination*. Human isolated Plts and PMN alone or Plts+PMN were incubated for 6 h with the indicated treatments, and cell-free supernatants were collected. The cytokines were measured by commercial ELISAs (eBioscience, San Diego, CA, USA) according to the manufacturer’s instructions.

### Mice

BALB/c mice were bred in the animal facility of the Institute of Experimental Medicine, Academia Nacional de Medicina, Buenos Aires. Male mice aged 9–16 weeks and weighing 20–25 g were used throughout the experiments. They were maintained under a 12-h light–dark cycle at 22 ± 2°C and fed with a standard diet and water *ad libitum*. The experiments performed were conducted according to principles set forth in the Guide for the Care and Use of Laboratory Animals (National Research Council, (1996)).

### Murine Model of HUS

Murine model of HUS was induced as previously described (Alves-Rosa et al., 2001). Briefly, mice received 1 ng/mouse of Stx2 in pyrogen-free saline 0.2% FBS *via* intravenous (i.v.) injection in the retro-orbital plexus 1 h after receiving intraperitoneally (i.p.) 1 µg/mouse of LPS. All mice were sacrificed before 72 h.

### Platelet Depletion

Platelet depletion protocol was performed as previously described (Dran et al., 2002). Plts-rich plasma from control mice was obtained and washed twice. Adult rabbits were injected (i.v.) with a suspension of 1×10^9^ Plts per dose on days 1, 10, 20, and 30. On day 40, rabbits were bled by puncture of the ear vein, and serum was maintained at −20°C. Rabbits were boosted monthly. In preliminary experiments, we determined that an i.p. injection of 100 µl of a 1:2 dilution of the antiserum induced an almost complete depletion of mice Plts 4 h after injection (number Plts ≤ 80 × 10^3^ µl^−1^ for depleted mice; control mice ≈ 900 × 10^3^ µl^−1^). Antiserum was given daily, and the first dose was administered 4 h before LPS/Stx2 treatment.

### *In Vivo* NETs Digestion

Control and LPS/Stx2-treated mice received an i.v. injection of Micrococcal Nuclease S7 (Hoffmann-La Roche, Basilea, Suiza) (150 U in buffer containing 5 nM Ca^++^ and 5 mM Mg^++^) every 12 h. The first dose was given 1 h before LPS/Stx2 treatment.

### Blood Sample Collection

Blood samples were obtained from each mouse by retro-orbital punction and collected on citrated tubes. Plasma was obtained by centrifugation of the blood at 1,000 *g* for 20 min at 4°C.

### Measurement of von Willebrand Factor

von Willebrand factor (vWF) in plasma samples from mice was determined by ELISA following the manufacturer’s instructions (Dako, Glostrup, Denmark). Results were expressed in ng ml^−1^ using normal pooled plasma as standard (National Institute for Biological Standards and Control, UK).

### Statistical Analysis

Statistical differences among treatments were determined using paired one-way analysis of variance (anova) followed by Bonferroni post-test for multiple comparisons. A *p* < 0.05 was considered significant. All tests were carried out using Prism 8.0 (Graph Pad Software, La Jolla, CA).

## Results

### Stx2 Promotes the Netotic Activity of Plts Treated With LPS

Previous reports have documented that Plts activated with a high dose of LPS induce netosis on PMN (Clark et al., 2007). In this work, we used a lower dose of LPS, which did not induce the release of NETs *per se*, as we seek to determine the contribution of Stx2 in the induction of netosis by Plts treated with LPS.

**Figure 1:**
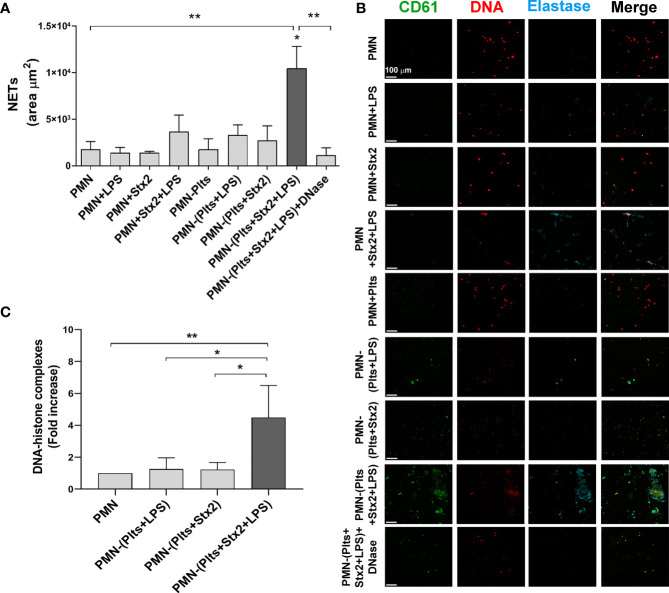
*Increased netosis mediated by Stx2 and LPS treated platelets.* Plts were left untreated (Plts), treated with Stx2 alone (20 ng ml^−1^) for 20 min, or treated also with LPS (0.3 µg ml^−1^) for 20 more min. After that, Plts were carefully washed and incubated with isolated autologous PMN. In addition, PMN were untreated (PMN) or treated directly with LPS and/or Stx2. Samples were seeded on poly-L-lysine treated glasses for 6 (h)** (A)** Area of NETs (µm^2^) was determined by fluorescence microscopy, labeling DNA with propidium iodide (PI), neutrophil elastase with an anti-elastase antibody, and Plts with an anti-CD61 antibody. (n = 5). *p < 0.01 vs. all other groups; **p < 0.005. **(B)** Micrographs from one representative independent experiment (200x). **(C)** NETs were also quantified by ELISA measuring DNA–histone complexes after releasing NETs onto supernatants by DNase controlled digestion. (n = 3). *p < 0.01; **p < 0.005. All results were expressed as the fold increase absorbance relative to unstimulated PMN (mean ± SEM).

Plts were treated as indicated in *Materials and Methods* and then were washed, incubated with isolated PMN from the same Plts donor, and left on poly-L-lysine-treated glasses for 6 h. NETs formation was determined using fluorescence confocal microscopy, labeling DNA fibers with propidium iodide (PI), elastase content with a specific antibody, and Plts with an anti-CD61 antibody. As control of NETs formation, DNase was used for total digestion of NETs. According to the area of NETs depicted in **Figures 1A, B**, LPS and/or Stx2 failed to induce NETs on PMN. Moreover, under our experimental conditions, Plts alone, LPS-treated, or Stx2-treated Plts did not induce significant netosis. However, when Plts were first stimulated with Stx2 before LPS, NETs were increased. Specific effects of Stx2 were determined on NETs formation by blocking the toxin with a specific anti-Stx2 antibody. As shown in **Supplementary Figure S1**, the addition of the anti-Stx2 decreased netosis.

To confirm the results observed by fluorescence microscopy, NETs were also quantified by the determination of DNA associated with histones (DNA–histone), after the release of NETs to the supernatant by controlled digestion using DNase. **Figure 1C** shows higher DNA–histone complexes when LPS-treated Plts were first stimulated with Stx2.

These results show that Stx2 sensitizes LPS-treated Plts to promote netosis.

### Stx2 and LPS Binding to Plts Are Increased by Stx2 Treatment

**Figure 2:**
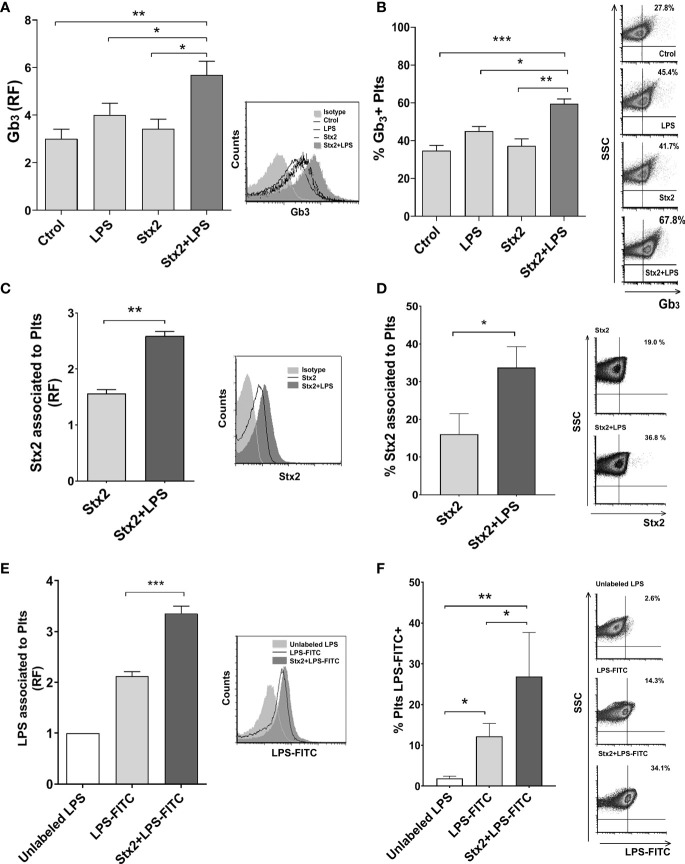
Stx2 and LPS treatment increased Gb_3_ expression and binding of Stx2 and LPS to Plts. Isolated Plts were left untreated (Ctrol) or treated with Stx2 (20 ng ml^−1^) for 20 min before addition or not of LPS (0.3 µg ml^−1^) for another 20 min (LPS or Stx2+LPS). Evaluation of Gb_3_ expression **(A)** and the percentage (%) of Gb_3_ expressing Plts **(B)** was performed by flow cytometry analysis. **(A)** Gb_3_ expression was expressed as the relative fluorescence (RF) of the mean fluorescence intensity (MFI) obtained on treated samples compared to isotype control. Histogram plots from one representative experiment are presented (right). (n = 4). **(B)** Percentage (%) of Gb_3_ expressing Plts. Density plots from one representative experiment are presented (right). (n = 4). **(C)** Stx2 association to Plts was evaluated on Plts treated with 100 ng ml^−1^ Stx2 for 5 min before adding LPS (0.3 µg ml^−1^) for another 20 min (Stx2 or Stx2+LPS). Stx2 binding was revealed using a specific anti-Stx antibody and analyzed by flow cytometry. Results were expressed as the RF of the MFI of treated Plts relative to untreated Plts. Histogram plots from one representative experiment are presented (right). (n = 3). **(D)** Percentage of Plts that have bound Stx2. Representative dot plots are shown on the right (n = 3). **(E)** Plts were treated with 20 ng ml^−1^ of Stx2 (Stx2) for 20 min before adding or not adding FITC-labeled LPS (2.5 µg ml^−1^) for another 20 min (LPS-FITC or Stx2+LPS-FITC). A control of unspecific FITC binding was established by adding 10 µg ml^−1^ of unlabeled LPS to a duplicate of treated-Plts. Fluorescence was determined by flow cytometry, and results were expressed as the RF of the MFI of treated Plts relative to unlabeled LPS exposed Plts. Histogram plots from one representative experiment are presented (right) (n = 3). **(F)** The percentage (%) of LPS-FITC + Plts was determined as in panel **(D)** and is shown. Density plots from one representative experiment are shown (right) (mean ± SEM). *p < 0.05; **p* < *0.005; ***p < 0.001.

In an attempt to elucidate the mechanism by which Stx2 promotes netosis in LPS-treated Plts, we first determined whether the toxin receptor, globotriaosylceramide (Gb_3_), which was previously described to be present in Plts (Ghosh et al., 2004), was modulated on Plts after stimulation with Stx2 and/or LPS. Analysis from flow cytometry studies shown in **Figure 2** reveal that only Stx2 combined with LPS was able to significantly increase both, Gb_3_ expression on Plts surface (**Figure 2A**), and the % of Gb_3_ expressing Plts (**Figure 2B**). To determine if this enhanced Gb_3_ expression correlated with an increased toxin binding, we evaluated Stx2 association on treated Plts using an anti-Stx2 antibody and analyzed the results by flow cytometry. As shown in **Figures 2C, D**, a significant increase in the level of toxin association was observed for Stx2+LPS-treated Plts compared to Stx2 alone stimulated Plts.

Given the results obtained so far, we decided to evaluate if the binding of LPS to Plts stimulated with Stx2 was modulated. FITC-labeled LPS was added to untreated or Stx2-treated Plts. A high dose of unlabeled LPS was then added to a pool of treated Plts to compete and displace FITC-LPS bound to Plts and establish unspecific/soluble FITC that may have associated unspecifically to Plts. Flow cytometry analysis depicted in **Figures 2E, F** demonstrated that Stx2 increases LPS binding to Plts compare to unstimulated Plts.

Taken together, these results suggest that direct binding of Stx2 or LPS occurs when both toxins are present.

### Stx2 Induces Activation of LPS-Stimulated Plts and Promotes PMN–Plts Mixed Aggregates Formation With PMN Activation

**Figure 3:**
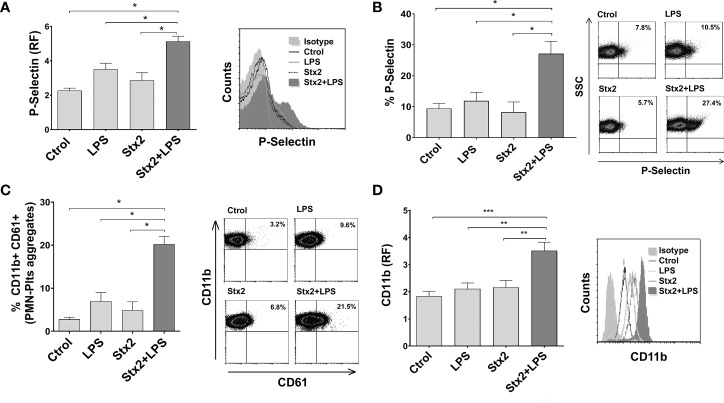
Increased Plts activation, PMN–Plts mixed aggregates formation, and PMN activation after Plts stimulation with Stx2 and LPS. **(A)** Plts were left untreated (Ctrol) or treated with 20 ng ml^−1^ Stx2 (Stx2) for 20 min before adding or not adding LPS (0.3 µg ml^−1^) for another 20 min (LPS or Stx2+LPS). Expression of P-selectin was determined by flow cytometry, and results of P-selectin fluorescence relative to isotype control (RF) are depicted. Histogram plots from one representative experiment are presented (right) (n = 3). **(B)** Percentage of P-selectin-positive Plts after treatment with the different stimuli. Representative dot plots are shown on the right (n = 3). **(C)** Treated Plts were washed to remove stimuli and then co-incubated with PMN for 10 min in a 1:50 PMN/Plts ratio. Cells were stained with PE-CD11b and FITC-CD61. The percentage of PMN–Plts mixed aggregates (CD11b+ CD61+) was analyzed by flow cytometry. Representative analysis of an independent experiment showing density plots is shown (right) (n = 3). **(D)** Treated Plts were washed and then co-incubated with PMN for 40 min in a 1:50 PMN/Plts ratio. CD11b expression was measured on PMN. Results are shown as the fluorescence of CD11b relative to isotype control (RF). Histogram plots from one representative experiment are presented (right) (n = 7) (mean ± SEM). *p < 0.05; **p < 0.005; ***p < 0.001.

Plts activation reflects a pro-thrombotic state, which is characteristic in HUS. Moreover, it has been suggested that direct binding of Stx occurs on activated Plts rather than on resting Plts (Ghosh et al., 2004). Considering this and given the results obtained in **Figure 2**, we decided to evaluate Plts activation by measuring P-selectin expression after Stx2 and LPS treatments on isolated Plts. As shown in **Figures 3A, B**, Stx2 pretreatment induced an increment in the percentage of P-selectin-positive Plts and its expression on LPS-stimulated Plts compared to untreated Plts, or Plts treated with LPS or Stx2 alone. The addition of an anti-Stx2 antibody decreased the increased P-selectin observed after the combined treatment of LPS+Stx2 (**Supplementary Figure S2**).

It has been previously described that Plts bind to PMN, triggering the formation of NETs (Clark et al., 2007; Carestia et al., 2016), and PMN and Plts activate each other in a cell–cell contact manner (Smyth et al., 2009). Therefore, we determined if Stx2 was able to promote PMN–Plts aggregates formation and PMN activation. Plts were treated and co-incubated with PMN, and cells were stained with anti-CD61 and CD11b fluorescent antibodies in order to identify Plts and PMN, respectively. PMN–Plts mixed aggregates (CD11b+ CD61+) were identified considering the presence of CD61+ events on CD11b+ gated PMN, to recognize Plts associated with PMN. As shown in **Figure 3C**, the percentage of PMN–Plts mixed aggregates was increased when Plts were treated with Stx2 and LPS compared to untreated Plts and LPS or Stx2 alone stimulated Plts. Moreover, **Figure 3D** depicts that the expression of CD11b on PMN was augmented when treated Plts were incubated with PMN. Again, these effects were abolished when an anti-Stx2 antibody was used (**Supplementary Figure S2**).

In summary, these results indicate that Stx2 promotes LPS-treated Plts to activate and aggregate with PMN resulting also in their activation.

Finally, in order to determine inflammatory cytokine secretion, IL-8 and IL-6 were evaluated. As observed in **Supplementary Figure S3**, both cytokines were increased only when Plts, PMN, or Plts+PMN were stimulated with LPS. The addition of Stx2 had no effect on IL-8 or IL-6 secretion. Neither anti-Stx2 treatment nor disruption of netosis by DNase modulate cytokine levels.

### Mixed PMN–Plts Aggregates Induced by Stx2 and LPS Promote Netosis *via* P-Selectin

**Figure 4:**
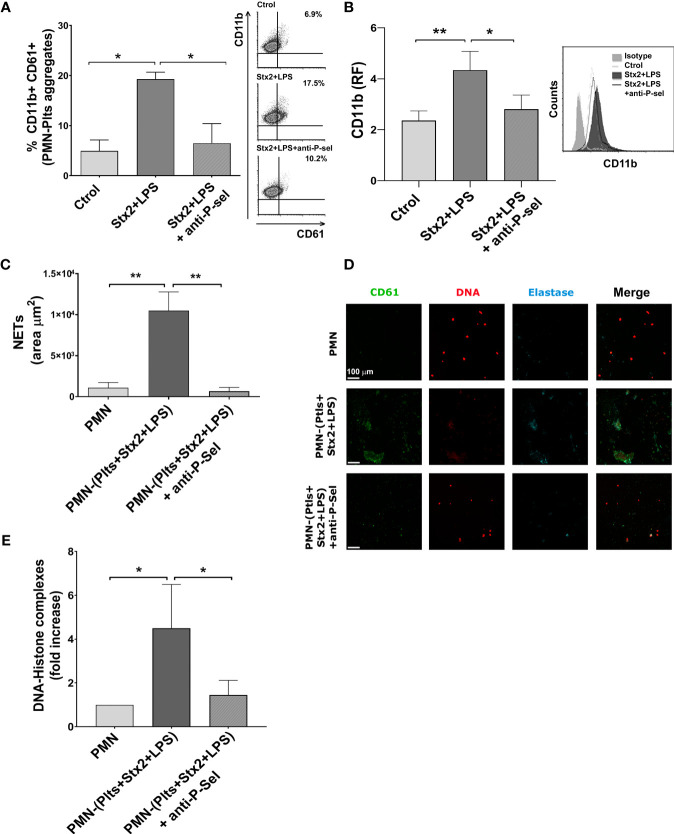
P-selectin blockade reduces mixed PMN–Plts aggregates formation, PMN activation, and netosis. Treated Plts were washed and then co-incubated with PMN in a 1:50 PMN/Plts ratio for 10 min **(A)**, 40 min **(B)**, and 6 h **(C–E)** in the presence or absence of an anti-P-selectin antibody. **(A)** The percentage of PMN–Plts mixed aggregates formation was determined by flow cytometry as the percentage of CD61+ Plts within the gate of CD11b+ PMN (% CD11b+ CD61+). Representative analysis of an independent experiment showing density plots is shown (right) (n=3). **(B)** CD11b expression on PMN was measured by flow cytometry and is shown as the median fluorescence relative to isotype control (RF). Histogram plots from one representative experiment are presented (right) (n = 6). **(C)** Area of NETs (µm^2^) was determined by fluorescence microscopy, labeling DNA with propidium iodide (PI), neutrophil elastase content with an anti-elastase antibody, and Plts with an anti-FITC-CD61 antibody (n = 7). **(D)** Micrographs from one representative independent experiment. **(E)** Fold increase absorbance relative to unstimulated PMN of DNA–histone complexes determined by ELISA after releasing NETs onto supernatants by DNase controlled digestion (n = 3) (mean ± SEM). *p < 0.05; **p < 0.005.

Since it has been suggested that binding of activated Plts to PMN involves P-selectin and the activated β2-integrin CD11b/CD18 (Evangelista et al., 1999), and given the significant increase in the percentage PMN–Plts aggregation observed in this context, we seek to determine if blocking P-selectin may reduce NETs formation induced by Stx2 and LPS-treated Plts. For this purpose, Plts were treated with the different stimuli and incubated with a monoclonal blocking P-selectin antibody before co-cultivation with autologous isolated PMN. As shown in **Figures 4A, B**, blocking P-selectin on treated Plts avoided the formation of PMN–Plts mixed aggregates and prevented upregulation of CD11b on PMN.

Given the above results, we determine if preventing the formation of PMN–Plts aggregates by blocking P-selectin could also reduce netosis induced by Stx2 and LPS on treated Plts. We found that when P-selectin was blocked, netosis promoted by Stx2 and LPS-treated Plts was diminished (**Figures 4C–E**).

### NETs Promoted by Stx2 and LPS-Stimulated Plts Increased HMEC-1 Sensitivity to PMN-Mediated Toxicity

Direct PMN–Plts-mediated endothelial toxicity in the context LPS/Stx2 has not been accurately addressed before, and to our knowledge, the contribution of netosis in this process has not been documented. In order to shed light on this critical aspect, we designed an *in vitro* model where PMN-mediated endothelial toxicity in the context of Stx2/LPS and Plts was evaluated.

We first wanted to determine individual PMN or Plts contributions to endothelial cell damage in the presence of LPS and Stx2. For this purpose, isolated PMN or Plts were treated or not with LPS or/and Stx2 and then were seeded on a monolayer of human microvascular endothelial cells, HMEC-1, for 18 h. The percentage of cytotoxicity was determined by scoring the number of live attached cells by light microscopy (see *Materials and Methods*).

**Figure 5:**
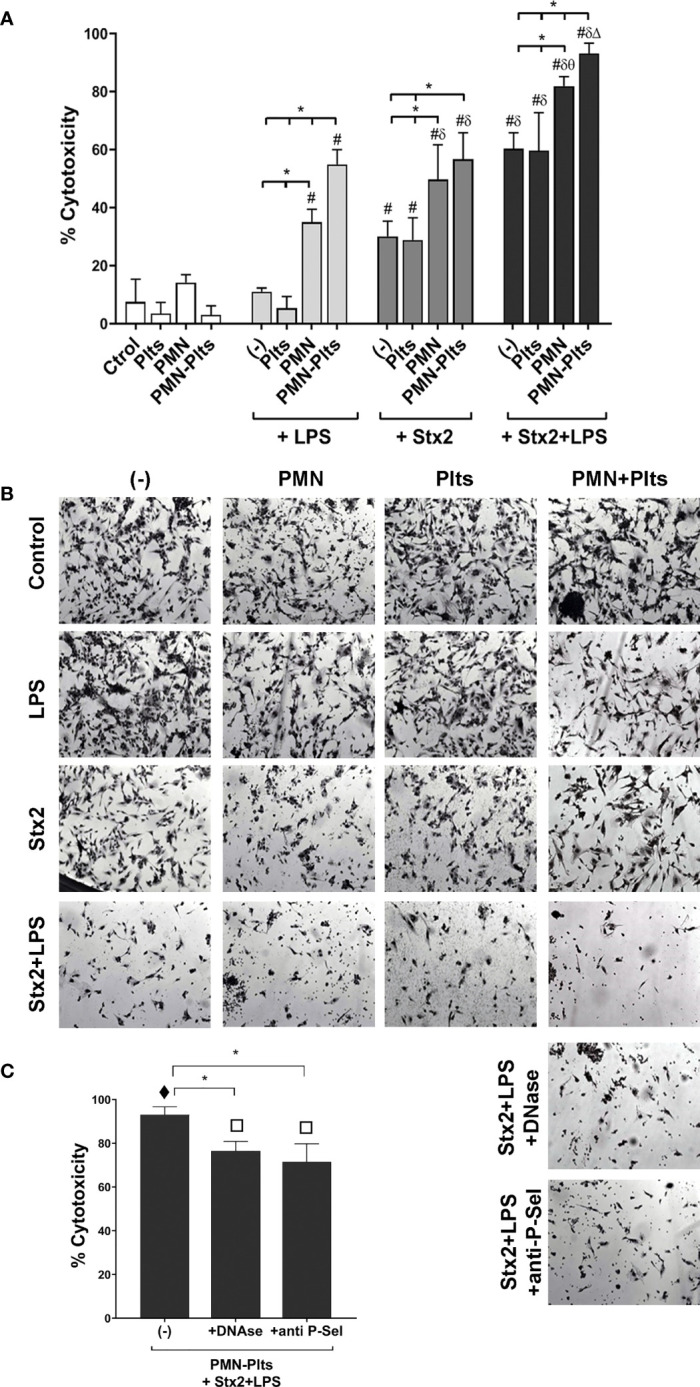
Relevance of netosis and mixed aggregates formation on endothelial cell damage. **(A)** HMEC-1 were cultured with 20 ng ml^−1^ Stx2 and/or 0.3 µg ml^−1^ LPS alone, untreated, or treated PMN (HMEC-1/PMN ratio, 1:20), or untreated or treated Plts (HMEC-1/Plts ratio, 1:1,000) for 18 (h) The percentage (%) of cytotoxicity was determined by light microscopy after carefully washing of detached cells and was calculated as described in *Materials and Methods* (mean ± SEM) (n = 5). **(B)** Micrographs of one representative independent experiment (200×). **(C)** Treated Plts were co-incubated with PMN (PMN/Plts ratio, 1:50) for 10 min in the absence or presence of DNase or anti-P-selectin blocking antibody. Cells were then seeded on HMEC-1 monolayers for 18 h, and the percentage of cytotoxicity was determined as in Panel **(A)** (mean ± SEM) (n = 5). *p < 0.05; ^#^ vs. Ctrol, Plts, PMN, PMN–Plts, LPS, LPS+Plts; ^δ^ vs. Stx2, Stx2+Plts; ^θ^ vs. LPS+PMN, Stx2+PMN; Δ vs. LPS+PMN–Plts, Stx2+PMN–Plts. ^τ^ vs. all other groups from Panel **(A)**; ^π^ vs. all other groups from Panel **(A)** except for PMN+Stx2+LPS.

**Figures 5A, B** shows that LPS did not induce endothelial damage compared to untreated cells. However, Stx2 increased the percentage of cytotoxicity, and this was even higher when Stx2 was combined with LPS. It is worth noting that the addition of Plts to LPS and/or Stx2-treated endothelial cultures failed to modify the observed toxicity, indicating that under our experimental conditions, Plts play no role in endothelial cell death. On the contrary, PMN induced endothelial toxicity when combined with LPS and potentiated Stx2-mediated toxicity compared to Stx2 alone. Moreover, LPS+Stx2-induced endothelial cell damage was increased in the presence of PMN.

Although Plts were not shown to potentiate endothelial toxicity in the context of LPS and Stx2, we evaluated the relevance of Plts on endothelial PMN-mediated toxicity. Plts were treated with LPS and Stx2 as before, then incubated with PMN for 10 min and seeded on HMEC-1 monolayers for 18 h. For this set of experiments, Plts were not washed in order to leave unbound/free Stx2 and LPS and let them impact endothelial cells in an attempt to better reflect an HUS scenario.

Additionally, **Figures 5A, B** reveals that combined PMN and Plts together with Stx2 did not modify endothelial toxicity compared with PMN in the presence Stx2 alone. However, and in line with our previous findings, maximal endothelial toxicity was observed when PMN were incubated with Plts in the context of LPS+Stx2. Based on this finding, we then determined the role of netosis and PMN–Plts aggregates formation in the maximal endothelial damage observed. DNase or an anti-P-selectin antibody were added to PMN+Plts before treatment, to degrade NETs and dissolve mixed PMN–Plts aggregates, respectively, before co-culturing them with HMEC-1. As can be observed in **Figure 5C**, DNase or anti-P-Sel reduced maximal endothelial cell toxicity, concluding that NETs formation and mixed aggregates formation are, in part, responsible for the endothelial damage in the context of LPS+Stx2. Controls of DNase or anti-P-Sel alone did not induce any cytotoxicity on HMEC-1 (data not shown).

In summary, these results highlighted the relevance of netosis in PMN-mediated endothelial cell damage in the context of LPS and Stx2, which is triggered by the formation of aggregates with Plts through P-selectin.

### Contribution of Plts in NET-Induced Endothelial Cell Damage in a Murine Model of HUS

We then decided to corroborate *in vivo* the relevance of NETs in endothelial damage using a mouse model of HUS and study, in particular, the role of Plts as promoters of netosis in this model. For this purpose, we conducted a series of experiments using an established murine model of HUS (Keepers et al., 2006).

Since our *in vitro* result indicated the relevance of PMN–Plts aggregates for netosis potentiation and endothelial damage in Stx2+LPS treatment, and given that HUS patients also show increased circulating aggregates (Ståhl et al., 2009), we first determine if mixed PMN–Plts aggregates were increased in LPS+Stx2-treated animals.

**Figure 6:**
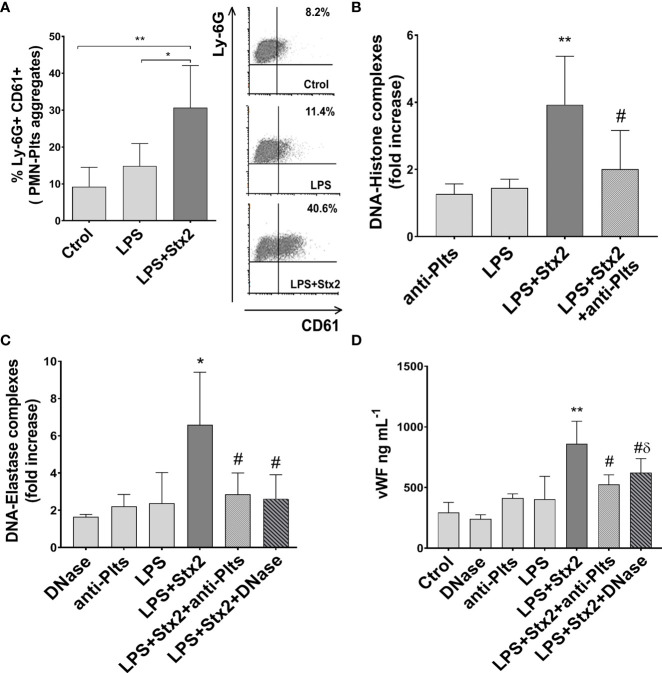
Mixed aggregates PMN–Plts, netosis, and endothelial dysfunction in LPS+Stx2-treated mice. Mice were treated with PBS (Ctrol), LPS (i.p.) alone or also with Stx2 (i.v.) 1 h after LPS. At 72 h, mice were anesthetized for bleeding before sacrificed. **(A)** Percentage of Ly-6G+ CD61+ mixed PMN–Plts aggregates measured in whole blood by flow cytometry. Representative analysis of an independent experiment showing density plots is depicted on the right (Ctrol n = 4, Stx2 n = 6, LPS+Stx2 n = 6). *p < 0.05; **p < 0.01. **(B)** NETs were detected in the plasma of treated mice using a DNA–Histone ELISA kit. For Plts depletion, mice received daily doses of a rabbit anti-mouse Plts immune serum. Results were expressed as the fold increase absorbance relative to PBS injected mice (anti-Plts n = 6, Stx2 n = 12, LPS+Stx2 n = 12, LPS+Stx2+anti-Plts n = 12). **p < 0.01 vs. anti-Plts and LPS; ^#^p < 0.05 vs. LPS+Stx2. **(C)** For *in vivo* NETs digestion, mice received an i.v. injection of DNase every 12 (h) DNA–elastase complexes were determined in plasma by ELISA. Results were expressed as the fold increase absorbance relative to PBS injected mice (DNase n = 6, anti-Plts n = 6, Stx2 n = 12, LPS+Stx2 n = 12, LPS+Stx2+anti-Plts n = 12, LPS+Stx2+DNase n = 12). *p < 0.05 vs. DNase, anti-Plts, and LPS; ^#^p < 0.05 vs. LPS+Stx2. **(D)** vWf plasmatic concentrations (ng ml^−1^) were determined in mice by ELISA (Ctrol n = 4, DNase n = 6, anti-Plts n = 6, Stx2 n = 12, LPS+Stx2 n = 12, LPS+Stx2+anti-Plts n = 12, LPS+Stx2+DNase n = 12) (mean ± SEM). **p < 0.01 vs. Ctrol, anti-Plts, DNase, and LPS; ^#^p < 0.05 vs. LPS+Stx2; ^δ^ p < 0.05 vs. DNase.

As shown in **Figure 6A** we observed an increase in the percentage of circulating PMN–Plts aggregates (Ly-6G+ CD61+) after 72 h of LPS and Stx2 inoculation. When NETs were determined by measuring circulating DNA–histone and DNA–elastase complexes (**Figures 6B,C**), only the LPS+Stx2 group showed a statistically significant increase compared to untreated or LPS alone treated mice. In addition, to confirm these data, a group of mice receiving Stx2 and LPS was also injected with DNase twice a day. **Figure 6C** shows a reduction in DNA–elastase levels as a consequence of DNase administration, confirming the formation of NETs in LPS+Stx2-treated mice.

Our *in vitro* results indicated that Stx2 promoted LPS-treated Plts to become strong inducers of NETs. Therefore, to confirm the relevance of Plts in NETs formation in the HUS model, we depleted Plts from mice before treating them with LPS/Stx2. A reduction in DNA–histone and DNA–elastase complexes levels was found when LPS and Stx2 were injected in Plts-depleted mice (**Figures 6B, C**). This result indicates a pivotal role of Plts as NETinducers in the model of HUS.

Endothelial dysfunction is the most important factor in the microangiopathic process of HUS, and von Willebrand factor (vWF) has been widely proposed as a biomarker of endothelial damage/dysfunction (Blann, 2006). Considering this, we determined plasmatic vWF levels in treated mice. As shown in the results depicted in **Figure 6D**, vWF plasmatic levels increased significantly above untreated or LPS-treated groups when mice were treated with LPS+Stx2. To determine whether NETs were implicated in this endothelial dysfunction, vWF levels were also measured in mice treated also with DNase. **Figure 6D** shows a decrease in plasmatic vWF levels when mice received DNase treatment together with LPS and Stx2, although this level was still high compared to a DNase alone control. This suggests that NETs induced in the context of LPS and Stx2 are in part responsible for endothelial dysfunction, but other components might play a role in the global endothelial dysfunction.

Finally, to assess the contribution of Plts to endothelial damage, Plts-depleted mice were injected with LPS+Stx2, and vWF levels were measured. As shown in **Figure 6D** Plts-depleted mice receiving LPS+Stx2 had lower vWF plasmatic levels, indicating that Plts contribute to the observed endothelial damage induced by LPS and Stx2 treatments.

## Discussion

**Figure 7:**
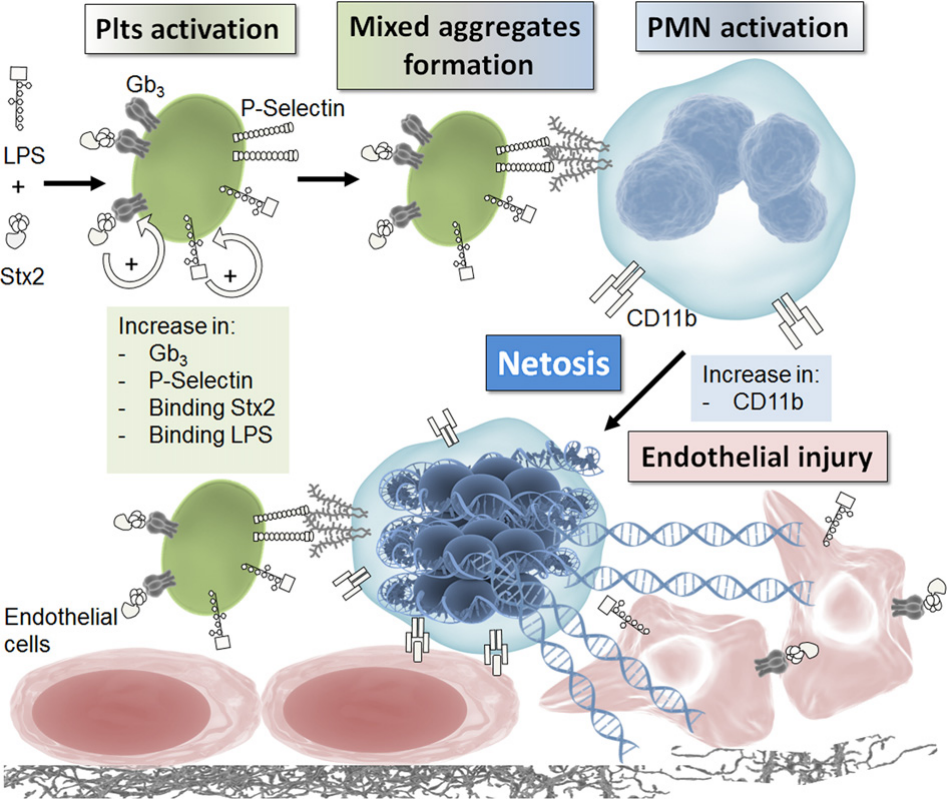
Schematic model. This model summarizes the results obtained in this work. See discussion for details.

In this study, we carry out a comprehensive study of the role of netosis in endothelial damage, integrating the main factors that could participate in this phenomenon in the context of HUS. **Figure 7** summarizes the results that emerge from this study. Both Stx2 and LPS were necessary to fully activate Plts and upregulate P-selectin to favor the interaction with PMN and mixed aggregates formation. As a consequence of this interaction, activation of PMN occurred and netosis was triggered, becoming an important factor that enhanced endothelial cell injury.

Our *in vitro* and *in vivo* findings describe for the first time a central role of Plts in NETs induction that directly contributes to endothelial cell injury in the context of HUS. It has been previously shown that Plts can serve as inducers of netosis, and therefore, the platelet–PMN interface is an important issue to be considered in order to understand the pathophysiology of HUS. Although some works have described the state of activation in Plts from HUS patients or the direct effect of Stx on Plts, we include LPS in our experimental settings, since it is well known that this bacterial derived component plays an important role in HUS pathogenesis (Paton and Paton, 1998), and anti-LPS antibodies are often found in HUS patients (Chart et al., 1998; Wijnsma et al., 2016). When Stx2 and LPS were used, Plts were able to trigger NETs as assessed by determination of DNA-histone and/or DNA-elastase complexes in isolated PMN–Plts assays or plasma from Stx2+LPS-treated mice. Additionally, we used DNase in order to break down NETs and confirmed both *in vitro* and *in vivo* the contributing role of this mechanism on endothelial damage.

Under our experimental conditions, neither LPS nor Stx2 alone, or combined, were able to directly induce NETs. Our treatment doses were lower compared to other reports that observed NETs induced by LPS alone (Caudrillier et al., 2012) or Stx alone (Ramos et al., 2016; Feitz et al., 2021), and this may account for the lack of NET induction using these mediators in the absence of Plts. Since we wanted to focus on the role of Plts as NET inducers in an HUS context, and evidence possible potentiating effects caused by Plts, in the first part of our study, we decide to use lower, non-netotic doses of LPS and Stx2 and also washed treated Plts before co-incubation with PMN to avoid the direct impact of free toxins on PMN. In this sense, our results indicate that Stx2 was lowering the amount of LPS necessary to make Plts effective NETs inducers. This central finding prompted us to investigate the mechanism by which Stx2 was potentiating the Plts-mediated stimulation of netosis. Previous reports have described the presence of Gb_3_ and also other Stx receptors on Plts and Stx binding to Plts (Tao et al., 1973; Cooling et al., 1998). In our experiments, we found an increase in the Gb_3_ receptor on Plts after treatment that was parallel with an increased Stx2 binding. This phenomenon has also been observed by us in astrocytes, where the combination of LPS and Stx increased Gb_3_ expression in levels higher than that found only with LPS and consequently increased Stx binding (Landoni et al., 2010). In this sense, it has been well documented that inflammatory factors, such as tumor necrosis factor alpha (TNF-α) and interleukin (IL)-1β, increase Gb_3_ in different Stx target cells (Van de Kar et al., 1992; Van De Kar et al., 1995; Ramegowda and Tesh, 1996). However, in this work, as observed in astrocytes, Stx2 itself creates a positive feedback loop, modulating its effects on Plts. Although the kidney, due to its high concentration in Gb_3_, is the central target organ of HUS, the modulation of Stx receptor expression caused by LPS or Stx itself could explain why other organs are also affected in some HUS patients (Khalid and Andreoli, 2019), and therefore, it would be interesting to study the impact of these molecules on the synthesis of sphingolipids for a deeper understanding of the pathogenesis of this disease.

In addition to the observed Stx2 association, in this study, we also found an increased LPS binding after Stx2 treatment. This is in line with the results of Ståhl, et al. who described that LPS binds to Plts through TLR4 and P-selectin (Ståhl et al., 2006). Moreover, these authors found LPS bound to Plts in HUS patients (Ståhl et al., 2006). The binding of LPS and Stx2 to Plts led to Plts activation as evidenced in this work and previously by others (Appiani et al., 1982; Rose et al., 1985; Chong et al., 1994; Karpman et al., 2001). In turn, this activation triggered the formation of PMN–Plts mixed aggregates (observed both *in vitro* and *in vivo*), an event considered to be a pre-requisite for subsequent NET formation (Caudrillier et al., 2012). In agreement with these results, increased mixed aggregates (Ståhl et al., 2009) and evidence of NETs in plasma (Ramos et al., 2016) have been found in HUS patients. In our work, we connected these two independent findings and described the importance of P-selectin in these events. In this regard, the upregulation of P-selectin by LPS and Stx2 was directly involved in the formation of mixed aggregates, causing PMN activation (CD11b upregulation) and triggering netosis, since blockade of the P-selectin molecule impaired all of these events. This result highlights the relevance of activated Plts, and P-selectin, on NET formation in the context of HUS. In line with this, we observed that *in vivo* depletion of Plts diminished plasmatic NETs in LPS+Stx2-treated mice. Reinforcing our finding, Etulain, et al. have also shown that thrombin activation of Plts induces mixed aggregates and netosis *via* P-selectin (Etulain et al., 2015). Further studies must be conducted to shed light on the potential use of therapies aimed to either reduce Plts activation, PMN–Plts aggregates formation, and therapeutic reduction of netosis for decreasing endothelial damage. In this sense, studies targeting platelet activation with either aspirin, a glycoprotein IIb/IIIa inhibitor, or a histone blocking antibody and DNase1 decreased NETs formation and lung injury in an experimental transfusion-related acute lung injury model (Caudrillier et al., 2012; Caudrillier et al., 2012).

In this work, we have demonstrated that NETs were in part responsible for the endothelial damage, as observed *in vitro* where DNase or an anti-P-selectin antibody reduced HMEC-1 cytotoxicity. In addition, LPS+Stx2 mice showed a reduced endothelial damage (assessed by vWf plasmatic levels) by the concomitant DNase treatment, or by Plts depletion, indicating that endothelial injury mediated by NETs depends on the presence of Plts. A complete reversal of cytotoxicity was not expected, since other mechanisms of damage mediated by PMN are not affected by DNase. In this regard, reactive oxygen species have been reported to play a major role in injury, and other PMN products, released from granules during activation, also contribute to cellular and tissue damage (Brown et al., 2006; Gabelloni et al., 2013) and cause disruption of the integrity of the endothelial barrier (Fox et al., 2013). In fact, endothelial damage mediated by the release of the granular content and its proteases and the production of toxic oxygen metabolites have been previously described as pathogenic mechanisms mediated by PMN in HUS (Forsyth et al., 1989; Gomez et al., 2013). Moreover, free or Plts-associated Stx2 may be directly inducing endothelial cell death (Van Setten et al., 1997; Ramegowda et al., 1999).

For our *in vivo* studies, we used both Stx2 and LPS to reproduce HUS in mice, as both mediators are present and are important participants in HUS pathophysiology. Using this model, we determined not only the occurrence of netosis but also investigated the role of Plts in endothelial damage. In this sense, for the first time, we described NETs formation in the model of HUS, by measuring DNA–elastase and DNA–histone complexes. Mixed aggregates were also observed in mice treated with LPS+Stx2, and increments in vWf plasmatic levels, accounting for endothelial damage, were also observed. Increased levels of circulating vWf have been widely associated with endothelial cell damage in several clinical conditions (Boneu et al., 1975; Castillo et al., 1977; Lufkin et al., 1979; Giustolisi et al., 1984; Pottinger et al., 1989; Blann et al., 1992), and high levels of plasmatic vWf have been shown in HUS patients (Tsai et al., 2001) and after Stx injection in the greyhound model of HUS (Raife et al., 2004). Moreover, endothelial cells released vWf after stimulation with Stx (Nolasco et al., 2005). It is accepted that most of the vWf in plasma comes from endothelial cells. Although Plts also have vWf stored in their alpha granules and release of vWf may occur after platelet activation, its contribution to circulating levels of vWf is considered minor. Supporting this, Kanaji *et al.* showed trace amounts of circulating vWf in chimeric mice that only have vWf in platelets (Kanaji et al., 2012). Instead, it has been proposed that Plts provide high local concentrations of vWf at sites of vascular damage but remain bound to Plts after activation, not exchanging with the plasmatic pool (Blann, 1993; Lip and Blann, 1997). Additionally, plasmatic vWf levels do not correlate with established platelet markers such as beta thromboglobulin (Blann, 1997), and aspirin reduces beta thromboglobulin but has no effect on vWf levels (Green et al., 1981). Therefore, it is unlikely that plasmatic vWf levels in treated mice came from Plts. Besides, vWf decreased in DNase-treated mice, even though DNase treatment only disaggregate NETs, and may not alter the activation of endothelial cells or Plts in mice, supporting that vWf levels is reflecting endothelial damage mediated by netosis.

Moreover, the decreased levels of vWf observed in Plts-depleted mice reflect the detrimental role of Plts mediating endothelial damage through the induction of NETs. Again, in line with *in vitro* findings, the reduction in vWf levels was partial, since other NET-independent factors are probably causing endothelial damage in mice. These results are in line with several observations that indicate that anti-platelet medications reduce mortality in infections and sepsis (Akinosoglou and Alexopoulos, 2014).

Our study describes for the first time the critical role of Plts as inducers of netosis and their contribution on endothelial cell injury in HUS. Considering this, it would be interesting to perform studies to determine whether the modulation of Plts levels or activation may be useful for the treatment of HUS by controlling endothelial damage.

## Data Availability Statement

The original contributions presented in the study are included in the article/**Supplementary Material**. Further inquiries can be directed to the corresponding author.

## Ethics Statement

The studies involving human participants were reviewed and approved by Comite de la ANM. The patients/participants provided their written informed consent to participate in this study. The animal study was reviewed and approved by IMEX CICUAL.

## Author Contributions

VL: conception or design of the work, data collection, data analysis and interpretation, drafting the article, and critical revision of the article. JP: data collection and data analysis and interpretation. AC: data collection and data analysis and interpretation. LC: data collection, conducted experiments with animals, and data analysis and interpretation. MN: data collection, data analysis and interpretation, and drafting of the article. DG: data collection, conducted experiments with animals, and data analysis and interpretation. FW: data collection, conducted experiments with animals, and data analysis and interpretation. MS: data analysis and interpretation and critical revision of the article. PS: conception or design of the work, data collection, data analysis and interpretation, and critical revision of the article. GF: conception or design of the work, data analysis and interpretation, drafting the article, and critical revision of the article.

## Funding

This study was supported by grants from Agencia Nacional de Promoción Científica y Tencnológica (ANPCyT, PICT 2019-0429) and CONICET (PIP 11220200100370).

## Conflict of Interest

The authors declare that the research was conducted in the absence of any commercial or financial relationships that could be construed as a potential conflict of interest.

## Publisher’s Note

All claims expressed in this article are solely those of the authors and do not necessarily represent those of their affiliated organizations, or those of the publisher, the editors and the reviewers. Any product that may be evaluated in this article, or claim that may be made by its manufacturer, is not guaranteed or endorsed by the publisher.
