# Supplementary figures and images for: Neutrophil Extracellular Traps Induced by Shiga Toxin and Lipopolysaccharide-Treated Platelets Exacerbate Endothelial Cell Damage
Source: Front Cell Infect Microbiol. 2022 Jun 23;12:897019. doi: 10.3389/fcimb.2022.897019 (PMC9262415; doi:10.3389/fcimb.2022.897019)

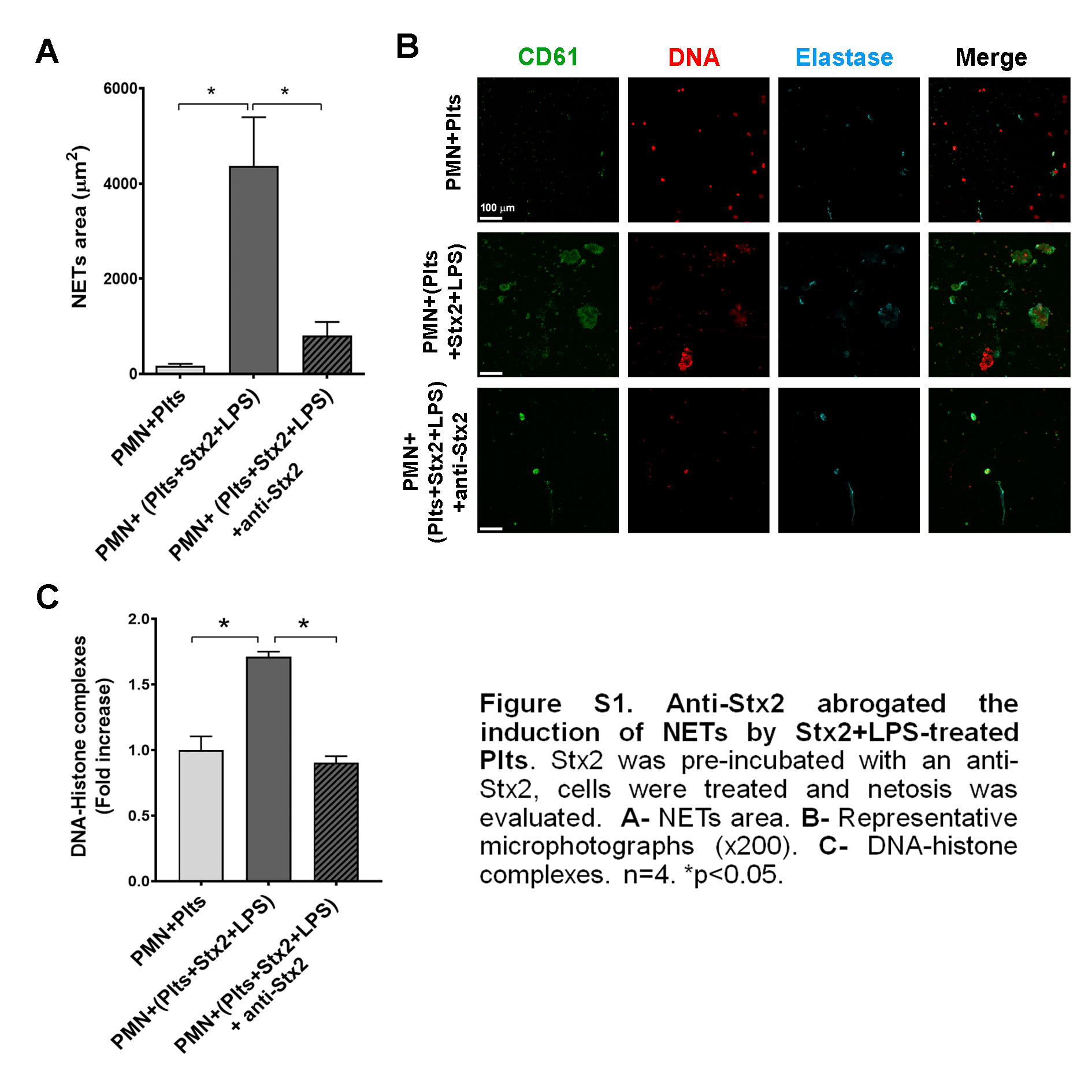

Supplement: Supplementary file 1 [file Image_1.tif]

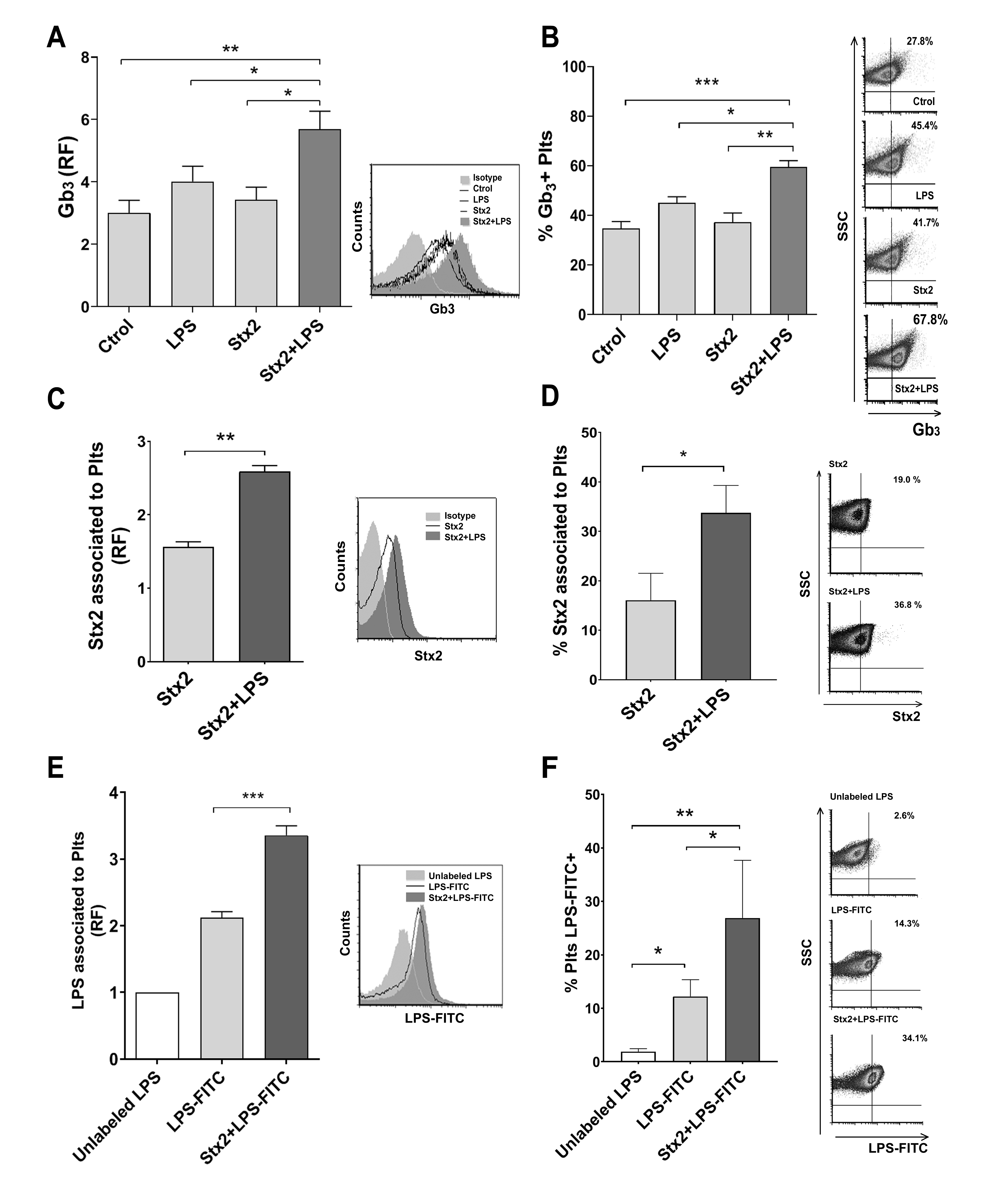

Supplement: Supplementary file 2 [file Image_2.tif]

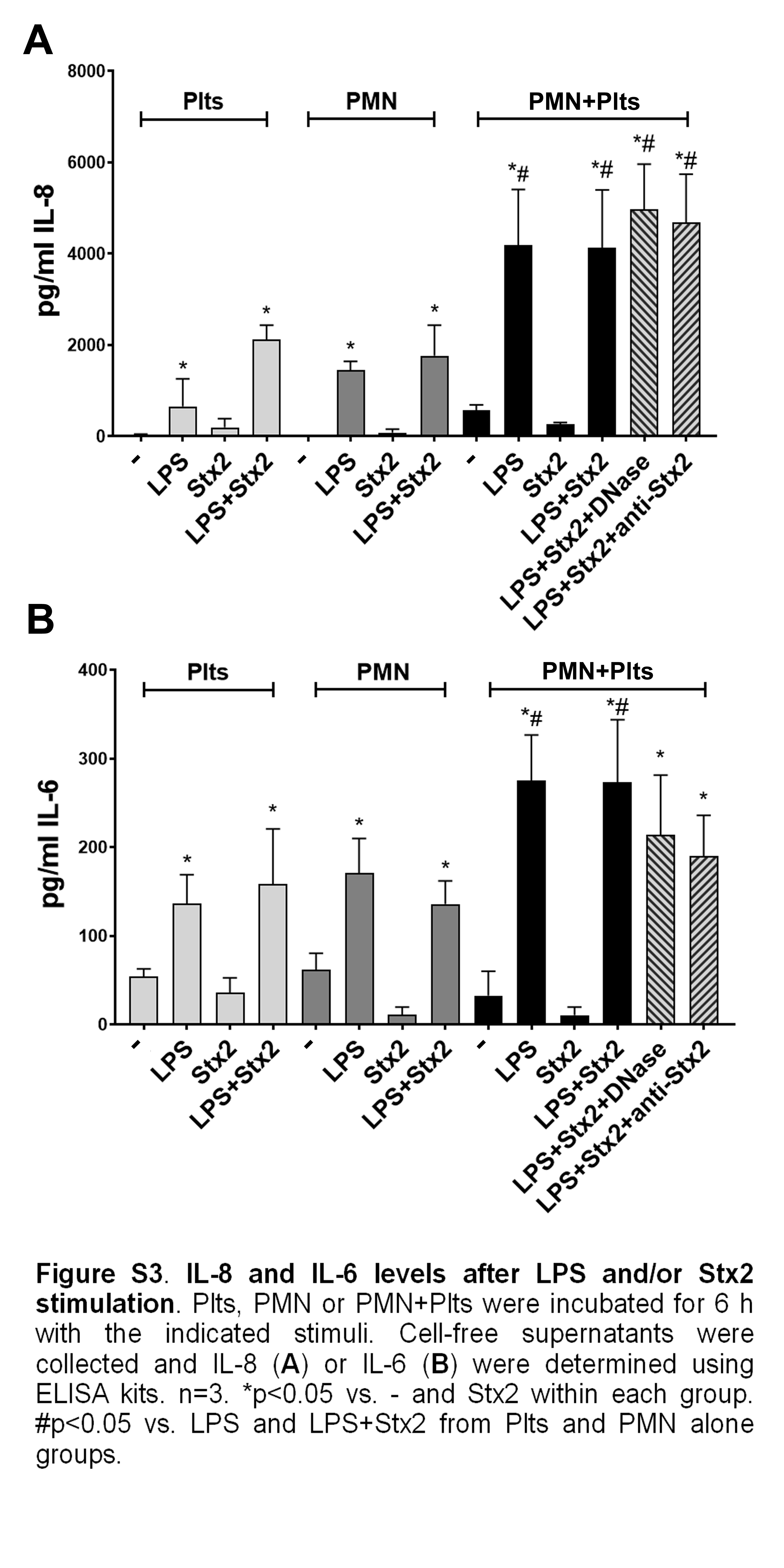

Supplement: Supplementary file 3 [file Image_3.tif]
